# Preliminary investigation of miRNA expression in individuals at high familial risk of bipolar disorder

**DOI:** 10.1016/j.jpsychires.2015.01.006

**Published:** 2015-03

**Authors:** Rosie May Walker, Joanna Rybka, Susan Maguire Anderson, Helen Scott Torrance, Ruth Boxall, Jessika Elizabeth Sussmann, David John Porteous, Andrew Mark McIntosh, Kathryn Louise Evans

**Affiliations:** a Medical Genetics Section, Centre for Genomic and Experimental Medicine, MRC Institute of Genetics and Molecular Medicine, The University of Edinburgh, Western General Hospital, Crewe Road, Edinburgh EH4 2XU, UK; b Division of Psychiatry, University of Edinburgh, Royal Edinburgh Hospital, University of Edinburgh, Edinburgh, UK; c Centre for Cognitive Ageing and Cognitive Epidemiology, The University of Edinburgh, 7 George Square, Edinburgh EH8 9JZ, UK

**Keywords:** Bipolar disorder, miRNA, Biomarker, Gene expression, PI3K/Akt, GABA

## Abstract

Bipolar disorder (BD) is a highly heritable psychiatric disorder characterised by recurrent episodes of mania and depression. Many studies have reported altered gene expression in BD, some of which may be attributable to the dysregulated expression of miRNAs. Studies carried out to date have largely studied medicated patients, so it is possible that observed changes in miRNA expression might be a consequence of clinical illness or of its treatment. We sought to establish whether altered miRNA expression might play a causative role in the development of BD by studying young, unmedicated relatives of individuals with BD, who are at a higher genetic risk of developing BD themselves (high-risk individuals). The expression of 20 miRNAs previously implicated in either BD or schizophrenia was measured by qRT-PCR in whole-blood samples from 34 high-risk and 46 control individuals. Three miRNAs, miR-15b, miR-132 and miR-652 were up-regulated in the high-risk individuals, consistent with previous reports of increased expression of these miRNAs in patients with schizophrenia. Our findings suggest that the altered expression of these miRNAs might represent a mechanism of genetic susceptibility for BD. Moreover, our observation of altered miRNA expression in the blood prior to the onset of illness provides hope that one day blood-based tests may aid in the risk-stratification and treatment of BD.

## Highlights

• Measured miRNA expression in unmedicated relatives of bipolar disorder patients.
• Identified miR-15b, miR-132 and miR-652 as being significantly up-regulated.
• Identified known and predicted targets of these miRNAs.
• Targets of these miRNAs are involved in functions implicated in bipolar disorder.

## 1 Introduction

Bipolar disorder (BD) is a severe psychiatric disorder characterised by recurrent episodes of mania and depression, which occurs with a lifetime prevalence of approximately 0.6% (Merikangas et al., 2011). The heritability of BD is estimated to be around 0.85 (McGuffin et al., 2003) and first-degree relatives of individuals with BD have a seven-fold risk of developing BD compared to the general population (Lichtenstein et al., 2009), indicating a substantial genetic component to the aetiology of this disorder. In addition to being at an elevated risk of developing BD, the first-degree relatives of BD patients are also at an increased risk of developing schizophrenia and major depressive disorder (MDD) (Lichtenstein et al., 2009; Rasic et al., 2014), indicating a shared component to the aetiology of these psychiatric disorders.

Altered gene expression has been identified in multiple studies of the blood and post-mortem brains of individuals with psychiatric disorders (Kumarasinghe et al., 2012; Seifuddin et al., 2013). Gene ontology analysis of these genes has implicated biological processes with apparent relevance to the pathogenesis of psychiatric illness such as synaptic function. More recently, altered microRNA (miRNA) expression has been identified as a putative pathogenic mechanism for both BD and schizophrenia (Maffioletti et al., 2014). Multiple miRNAs have been found to show dysregulated expression in individuals with these conditions both in the brain (Banigan et al., 2013; Beveridge et al., 2010; Miller et al., 2012; Moreau et al., 2011; Perkins et al., 2007; Santarelli et al., 2011; Smalheiser et al., 2014) and in the periphery (Gardiner et al., 2012; Lai et al., 2011). Consistent with these changes, increased expression of components of the miRNA processing pathway has been detected in the brains of schizophrenic patients (Beveridge et al., 2010; Santarelli et al., 2011). Additionally, the expression of Ago2, which is involved in effecting miRNA-induced silencing (Cenik and Zamore, 2011), as well as the regulation of mature miRNA expression and miRNA processing (Diederichs and Haber, 2007), has been found to be down-regulated in peripheral blood mononuclear cells in schizophrenic patients (Gardiner et al., 2013). miRNAs are short (∼22 nt) RNA sequences that can each target hundreds of mRNAs by complementarity to the 3′ untranslated region, generally resulting in the suppression of gene expression either by affecting mRNA stability or translation (Valencia-Sanchez et al., 2006). As such, changes in miRNA expression might underlie some of the observed changes in mRNA and protein expression observed in BD and schizophrenia.

Studies reporting altered miRNA expression in BD and schizophrenia have almost exclusively been carried out on individuals who have been ill for several years and are taking medication. It is, therefore, often not possible to determine whether observed changes in miRNA expression are a primary causative event, occur as a consequence of illness progression, or are induced by medication. Studies in primary neuronal cultures, cell lines and animal models have demonstrated that several miRNAs show altered expression following treatment with mood-stabilisers and/or antipsychotics (Chen et al., 2009; Gardiner et al., 2014; Hunsberger et al., 2013; Santarelli et al., 2013; Zhou et al., 2009), further supporting the need to study unmedicated individuals.

To our knowledge, only one study has measured miRNA expression in unmedicated individuals diagnosed with BD. Rong et al. (2011) measured a single miRNA, miR-134, and demonstrated reduced plasma expression in drug-free manic patients with BD1, which increased in response to treatment with mood-stabilisers.

We sought to further understanding of the involvement of altered miRNA expression in the aetiology of BD by measuring miRNA expression in young, unaffected relatives of patients with BD who are at higher genetic risk of developing BD (henceforth referred to as high-risk individuals). The high-risk and control individuals were selected from a larger cohort recruited as part of the Edinburgh-based Bipolar Family Study (BFS). Individuals from this cohort have undergone structural and functional magnetic resonance imaging, which has revealed reduced white matter integrity and alterations in the activity of the amygdala and insula, brain structures previously implicated in the pathophysiology of psychiatric illness, in high-risk individuals (Sprooten et al., 2011; Whalley et al., 2011, 2013b). Additionally, high-risk individuals have been shown to carry more BD-associated variants than control subjects, as indicated by a significantly higher BD polygene score (Whalley et al., 2013a).

Here, we measured the expression of 20 miRNAs previously implicated in the pathogenesis of BD and/or schizophrenia in whole-blood samples from high-risk and control individuals. As such, we aimed to identify miRNAs whose expression is altered prior to illness onset and which, thus, may play a causative role in illness development.

## 2 Methods

### 2.1 Participants

We examined 34 unaffected individuals at higher genetic risk of developing a mood disorder and 46 control subjects. These individuals were recruited as part of The Bipolar Family Study (BFS), as described previously (Sprooten et al., 2011; Whalley et al., 2011). High-risk subjects met the following selection criteria: (i) at least one first-degree or two second-degree relatives suffering from BD1; (ii) no personal history of BD; and (iii) aged 16–25 at the time of recruitment. Control individuals of a similar age were selected on the basis that they did not have a personal history of BD or a family history of any mood disorder amongst their first-degree relatives. Exclusion criteria for both groups were: a history of major depression, mania, or hypomania, psychosis, generalized anxiety disorder, panic disorder, eating disorder, substance dependence, an IQ < 70 or clinical diagnosis of learning disability, any major neurological disorder or history of head injury that included loss of consciousness, and any contraindications to magnetic resonance imaging (MRI). Only unrelated individuals were included. All participants provided written informed consent and the study was approved by the Multicentre Research Ethics Committee for Scotland.

### 2.2 Selection of miRNAs for assessment

MicroRNAs were selected for assessment on the basis that they were either implicated in the pathogenesis of BD or schizophrenia through: (i) altered expression in patients or (ii) altered expression in response to treatment with a mood-stabilising drug. A literature search was carried out using PubMed using the terms “micro RNA”, “bipolar disorder”, “schizophrenia”, “lithium”, and “valproate”, together with commonly used abbreviations, in August 2011.

### 2.3 Extraction of blood RNA

Blood for RNA extraction (3 ml) was collected in Tempus Blood RNA Tubes (Life Technologies). RNA was extracted using MagMAX for Stabilized Blood Tubes RNA Isolation Kit (Life Technologies), according to the manufacturer's instructions. RNA yield and quality were assessed using the Agilent Bioanalyzer.

### 2.4 Assessment of miRNA expression

Assessment of miRNA expression was carried out using TaqMan MicroRNA Assays (Life Technologies; SOM Table 1), following the manufacturer's protocol for custom reverse transcription and pre-amplification pools (https://tools.lifetechnologies.com/content/sfs/manuals/cms_094060.pdf). Briefly, for all genes of interest and reference genes, a custom reverse transcription (RT) pool was created by combining 10 ul of each 5× RT primer and a custom pre-amplification pool was created by combining 10 ul of each 20× TaqMan MicroRNA Assay. The RT reaction was performed using 100 ng RNA. A half volume reaction without the RT enzyme was also performed using 50 ng RNA (RT negative). cDNA was pre-amplified prior to use in quantitative reverse transcription polymerase chain reaction (qRT-PCR). qRT-PCR reactions were performed using an ABI Prism 7900 Sequence Detection System (Applied Biosystems). Expression levels were calculated using the relative standard curve method and miRNA-of-interest expression levels were normalised to the geometric mean of the expression levels of four small reference RNAs (RNU6B, RNU47, SNORD68, and SNORD95). These reference RNAs were selected from an initial pool of seven (RNU6B, RNU47, SNORD96A, SNORD61, SNORD68, SNORD95, and SNORD72) on the basis of geNorm analysis (Vandesompele et al., 2002). Each sample was measured in technical triplicate and the mean of this triplicate used in downstream analyses. Outlier samples, defined as data points falling outside of the range defined by median ± 3× inter-quartile range, were excluded.

### 2.5 Statistical analysis

Differences in age and RIN between the control and high-risk groups were assessed using two-tailed unpaired Student's t-tests. The between-group difference in gender was assessed using Fisher's exact test. In all cases a *p*-value of ≤0.05 was considered statistically significant.

Differences in gene expression between the control and high-risk subjects were assessed using two-tailed Mann–Whitney U tests. A *p*-value of ≤0.05 was considered to be nominally statistically significant. A Bonferroni correction was implemented to correct for multiple testing. The pairwise correlation between miRNA expression levels was assessed by Spearman's rank correlation coefficient.

### 2.6 Identification and functional analysis of miRNA targets

The regulatory targets of differentially expressed miRNAs were identified using Ingenuity Pathway Analysis's (IPA) miRNA Target Filter function, which queries TarBase and miRecords for experimentally validated miRNA targets and TargetScan for predicted miRNA targets. In addition, the Ingenuity Knowledge Base contains information on miRNA targets from the peer-reviewed literature.

The list of experimentally observed and predicted miRNA targets was submitted to IPA core analysis to identify canonical pathways (defined as manually curated, well-characterised metabolic and cell signalling pathways), diseases and biological functions that were enriched amongst the inputted list. IPA assesses enrichment using the right-tailed Fisher's exact test. As multiple hypotheses were tested, the Benjamini–Hochberg false discovery rate (FDR) method was implemented to calculate *q*-values. We defined statistical significance as *q* ≤ 0.05. The Ingenuity Knowledge Base was used as the reference set and the following parameter settings were used: experimentally observed direct and indirect relationships identified in all species.

## 3 Results

### 3.1 Sample demographics

Between-group differences in age, gender and RNA integrity, as represented by the RNA Integrity Number (RIN), were assessed as these factors can alter gene expression. No significant differences were observed (all *p* > 0.05; Table 1; SOM Table 2).

### 3.2 Selection of miRNAs for assessment

Twenty miRNAs were selected for assessment on the basis that they had previously been implicated in the pathogenesis of BD or the closely related condition, schizophrenia (SOM Table 3). Of these, 16 miRNAs (miR-134, miR-212, miR-133b, miR-449, miR-34a, miR548d-5p, let-7b, let-7c, miR-15b, miR-132, miR-145, miR-195, miR-221, miR-432, miR-572, and miR-652) were successfully detected in our whole-blood cDNA samples. miR-15a, miR-154*, miR-548d-3p, and miR-564 were not reliably detected in a sufficient number of samples to permit analysis.

### 3.3 Identification of differentially expressed miRNAs

**Fig. 1:**
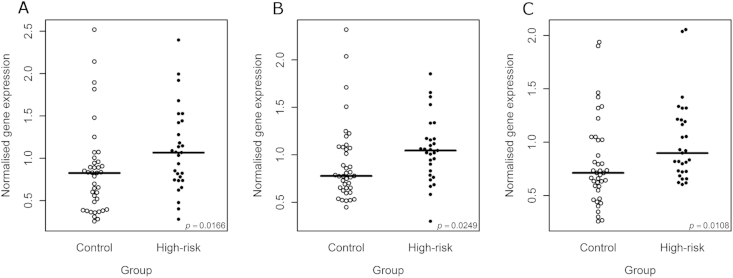
Beeswarm plots showing normalised expression values for miR-15b (A), miR-132 (B), and miR-652 (C) in high-risk and control individuals. The horizontal bar indicates the median expression value. The *p*-value in the bottom right-hand corner of each plot represents the significance (uncorrected) of the Mann–Whitney U test.

**Fig. 2:**
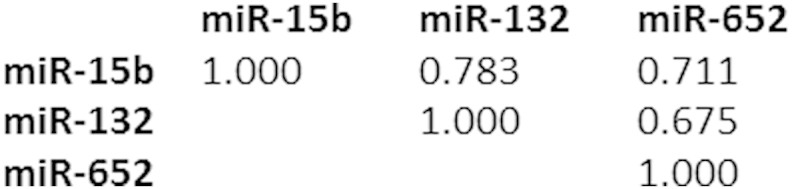
Spearman's rank correlation coefficients for the three nominally differentially expressed miRNAs, miR-15b, miR-132 and miR-652.

Of the sixteen reliably detected miRNAs, three, miR-15b, miR-132, and miR-652, showed a nominally significant increase in expression in the high-risk subjects compared to the control subjects (miR-15b: *p* = 0.0166, fold-change (FC) = 1.29; miR-132: *p* = 0.0249, FC = 1.34; miR-652: *p* = 0.0108, FC = 1.26; Fig. 1; Table 2). None of these changes remained significant after implementing a Bonferroni correction for multiple testing (all *p* ≤ 0.172). The expression levels of all three dysregulated miRNAs were, however, positively correlated (Fig. 2).

### 3.4 Pathway analysis of the targets of differentially expressed miRNAs

To identify biological pathways that might be affected by the differential expression of miR-15b, miR-132, and miR-652 we carried out pathway analysis. IPA's miRNA Target Filter function was used to generate lists of experimentally observed and predicted targets of the three dysregulated miRNAs. miR-15b was found to have 2020 targets (187 experimentally observed, 1833 predicted), miR-132 was found to have 767 targets (9 experimentally observed, 758 predicted), miR-652 was found to have 205 targets (all predicted). The results of these analyses are summarised in Tables 3–5.

Consistent with their proposed role in psychiatric illness, the targets of both miR-15b and miR-132 were enriched for molecules involved in Nervous System Development and Function (miR-15b: *q* = 1.92 × 10^−8^–1.50 × 10^−2^; miR-132: *q* = 4.98 × 10^−8^–2.83 × 10^−3^). For both miRNAs, PI3K/Akt Signalling (miR-15b: *q* = 4.13 × 10^−7^; miR-132: *q* = 1.17 × 10^−5^) was amongst the top canonical pathways and the related PTEN Signalling pathway was amongst miR-15b's top canonical pathways (*q* = 1.51 × 10^−7^). The targets of miR-132 were found to be enriched for molecules in the IPA category Neurological Diseases (*q* = 1.23 × 10^−7^–2.71 × 10^−3^). Within this category, the most significant sub-classification was Cognitive Impairment (*q* = 3.03 × 10^−5^), followed by Mental Retardation (*q* = 1.38 × 10^−4^). The Neurological Diseases sub-classification Schizophrenia was also enriched for miR-132's target mRNAs (*q* = 1.65 × 10^−2^). Additionally, Axonal Guidance Signalling was a top canonical pathway for the targets of miR-132 (*q* = 7.03 × 10^−^7).

Pathway analysis of miR-652's targets did not yield any results that survived multiple testing correction. Canonical pathways attaining nominally significant *p*-values included Reelin Signalling in Neurons (*p* = 8.88 × 10^−4^) and GABA Receptor Signalling (*p* = 3.64 × 10^−3^). Amongst the diseases showing nominally significant enrichment for the targets of miR-652 was the category of Neurological Diseases (*p* = 2.61 × 10^−4^–4.61 × 10^−2^). Within this category, the sub-classifications of Schizoaffective Disorder (*p* = 1.84 × 10^−3^) and Bipolar Disorder (*p* = 7.10 × 10^−3^) were significantly enriched.

## 4 Discussion

In recent years, it has become clear that changes in miRNA expression are likely to play a role in the pathogenesis of psychiatric disorders, and have the potential to act as peripheral biomarkers of disease and markers of therapeutic response (Fan et al., 2014; Lai et al., 2011; Maffioletti et al., 2014; Rong et al., 2011). Most studies carried out to date have focussed on the role of miRNAs in schizophrenia; however, studies in patients with BD have also found evidence for altered function (Kim et al., 2010; Moreau et al., 2011; Rong et al., 2011; Smalheiser et al., 2014). These studies have, however, almost exclusively studied medicated individuals who have been ill for some time, leaving open the possibility that observed changes in miRNA expression are a consequence of illness progression or treatment.

The need to establish whether altered miRNA expression plays a causal role in the development of BD prompted us to study a group of individuals who, due to family history, are at a higher genetic risk of developing BD than the general population. The expression of 20 miRNAs, selected for *a priori* evidence of involvement in BD or schizophrenia, was measured in these high-risk individuals and compared with a control group. Three miRNAs, miR-15b, miR-132 and miR-652, which have previously been implicated in schizophrenia, show nominally significant increases in expression. Although our findings do not remain significant after implementing the Bonferroni correction, it is important to note that the highly correlated expression of the miRNAs assessed here will render this correction overly conservative. The resultant inflation in type 2 error rate is likely to be further compounded by the fact that each miRNA was selected based on *a priori* evidence for the involvement of its altered expression in bipolar disorder and/or schizophrenia. Although replication is essential, our findings provide suggestive evidence that the dysregulated expression of these miRNAs might represent a mechanism underlying genetic susceptibility for BD.

In keeping with findings in patients with schizophrenia, the expression of all three miRNAs was increased in the high-risk subjects. miR-15b was previously found to show increased expression in the superior temporal gyrus (STG) and dorsolateral prefrontal cortex (DLPFC) of patients with schizophrenia (Beveridge et al., 2010). miR-132 has been found to show altered expression in schizophrenia patients in two studies; however, they disagree with regard to the direction of change in miR-132's expression (Kim et al., 2010; Miller et al., 2012). Both studies measured miR-132 expression in the prefrontal cortex (PFC); however, Kim et al. (2010) observed increased expression in schizophrenia while Miller et al. (2012) observed decreased expression in two independent cohorts of patients with schizophrenia. An increase in the expression of miR-652 expression has been observed in the DLPFC of patients with schizophrenia (Santarelli et al., 2011). Interestingly, miR-652 was one of seven miRNAs found to form a blood-based expression signature that was able to discriminate between patients with schizophrenia and control subjects with an area under the curve of 0.85 (Lai et al., 2011); increased miR-652 expression was observed in these patients.

miR-15b is a member of a family of miRNAs that are implicated in a wide range of functions and diseases including metabolism, angiogenesis, stress response, cancer, cardiovascular disease and neurodegenerative conditions, including Alzheimer's disease (Finnerty et al., 2010). Decreased miR-15b expression forms part of a seven miRNA plasma expression signature that was found to distinguish patients with Alzheimer's disease from controls with greater than 95% accuracy (Kumar et al., 2013). miR-132 is transcribed from a cluster of miRNAs that are known to play an important role in neuronal development and function (Wanet et al., 2012) and decreased miR-132 expression has been observed in Alzheimer's disease (Cogswell et al., 2008). Fewer published studies have explored the functions of miR-652; however, it appears to play a central role in myeloid development and, thus, innate immunity (Gilicze et al., 2014). There is evidence linking altered innate immune function to the pathogenesis of BD (Jones and Thomsen, 2013), suggesting one pathway by which altered miR-652 expression might confer risk for BD. Without sorted cell populations, we cannot determine whether this change in expression reflects a change in whole blood composition; however, should our findings be replicated, it would be important to assess this possibility.

Our observation of positive correlations between the three dysregulated miRNAs is compatible with a scenario in which a common miRNA regulatory or processing mechanism has been perturbed in individuals at high-risk of developing BD. Previous studies have shown expression of components of the miRNA processing pathway to be dysregulated in patients with schizophrenia (Beveridge et al., 2010; Gardiner et al., 2013; Santarelli et al., 2011). Investigating whether aspects of the miRNA processing pathway are altered in the high-risk cohort would be of interest.

To gain an insight into the particular biological processes that might be affected by the altered expression of miR-15b, miR-132 and miR-652 expression, we performed pathway analysis of the experimentally observed and predicted targets of these miRNAs. Of particular interest was the fact that the targets of miR-15b and miR-132 were found to be involved in PI3K/Akt signalling both directly and, in the case of miR-15b, indirectly via the modulation of PTEN, a negative regulator of PI3K/Akt signalling. PI3K/Akt signalling is involved in several processes that are perturbed in psychiatric patients, including mediation of the neuroprotective effects of neurotrophins, such as brain-derived neurotrophic factor (BDNF), and the induction of long-term plasticity, which is required for learning and memory (Zheng et al., 2012). Activation of the PI3K/Akt signalling pathway results in the inhibition of glycogen synthase kinase-3 (GSK3). GSK3 signalling has been implicated in psychiatric illness by multiple lines of genetic and functional evidence and the inhibition of GSK3β is believed to be one of the main mechanisms of action of the mood-stabilising drug lithium (Kitagishi et al., 2012).

The targets of miR-132 were found to be enriched for molecules involved in cognitive function. Diminished cognitive ability is a well-established phenomenon in BD (Raust et al., 2014) and has also been observed in the first-degree relatives of individuals with BD (Nehra et al., 2014).

Although none of the pathway analysis results for miR-652's targets withstood correction for multiple testing, it was of interest to note that the top-ranked canonical pathways and diseases were pertinent to BD. miR-652's targets were nominally significantly enriched for molecules implicated in BD and schizoaffective disorder. Amongst these targets were the gamma-aminobutyric acid (GABA) receptor subunits GABARB2 and GABARB3, the serotonin receptor 5-HT1D, and Disrupted in Schizophrenia 1 (DISC1). GABAergic interneuron dysfunction is believed to play a central role in the pathogenesis of schizophrenia (Nakazawa et al., 2012) and the interaction between DISC1 and GABA signalling has been shown to regulate neurogenesis in mice (Kim et al., 2012). miR-652's targets were also nominally significantly enriched for molecules involved in Reelin signalling. Reelin signalling contributes to multiple processes relevant to the pathogenesis of schizophrenia and BD: in development, it is involved in regulating neuronal migration and brain lamination and in the adult brain it modulates synaptic plasticity (Folsom and Fatemi, 2013). While there are a small number of negative studies, the majority of gene expression and genetic association studies implicate altered Reelin signalling in schizophrenia and BD (Folsom and Fatemi, 2013). These findings must, of course, be interpreted with the caveat that they failed to attain statistical significance after correcting for multiple testing. This may, in part, reflect the fact that relatively little is known about miR-652's function, resulting in a much shorter list of interactors being available for pathway analysis.

Taken together, the results from our pathway analyses and the published literature suggest several neurological systems that might be impacted by the dysregulated expression of miR-15b, miR-132 and miR-652, thus conferring risk for BD. Whilst the evidence linking the altered expression of these miRNAs with biological pathways previously implicated in the pathogenesis of BD is compelling, it is important to note some key limitations of the present study. The most important limitation is the size of the sample studied. This small sample size means that failure to observe a change in expression in the majority of the miRNAs measured cannot be taken as evidence that the altered expression of these miRNAs does not represent a mechanism of genetic risk for BD; it is possible that our study was simply underpowered to detect small, but real, changes in expression. Moreover, it is imperative that our finding of altered miR-15b, miR-132 and miR-652 expression is replicated in a larger independent sample.

The nature of our study design (i.e. young, unmedicated individuals at high genetic risk of BD) necessitated the measurement of gene expression in a non-neuronal tissue. While we do not know whether the changes in miRNA expression we observe in the blood of these individuals are also present in the brain, it is of interest that all three differentially expressed miRNAs have previously been found to show dysregulated expression in brain tissue from patients with schizophrenia in a direction consistent with our findings (Beveridge et al., 2010; Kim et al., 2010; Santarelli et al., 2011).

We measured miRNA expression in whole-blood, which is formed of multiple different cell types, each with its own characteristic miRNA expression profile. It is, therefore, possible that apparent changes in miRNA expression might reflect differences in the relative proportions of blood cell subtypes between the high-risk and control groups. An additional issue when measuring miRNA expression in whole-blood is the presence of cell-free miRNAs. The snoRNAs used to normalise miRNA expression in the current study are not likely to represent good normalisers for cell-free miRNAs, due to the low expression of snoRNAs in this blood component. However, compared with erythrocytes, granulocytes and platelets, cell-free miRNAs make up a small proportion of total miRNA expression in whole-blood RNA (Kang et al., 2012; Sangokoya et al., 2010). Therefore, it is unlikely that the changes in expression we observe are driven entirely by expression changes in cell-free blood. Nevertheless, it would be of interest to characterise the expression of miR-15b, miR-132 and miR-652 in different blood cell types and cell-free blood. It should, however, be noted that should a blood-based biomarker be developed to aid in the diagnosis of schizophrenia and/or bipolar disorder, the ability to measure this biomarker in whole-blood would vastly improve its utility.

For all three dysregulated miRNAs there is a substantial degree of overlap in expression between the control and high-risk individuals. This likely reflects both the existence of varying levels of predisposition in the high-risk group and the complex and heterogeneous nature of BD. It is possible that those individuals who do go onto become ill, or those who become ill and whose illness is characterised by particular features, show the most extreme changes in miRNA expression. Longitudinal follow-up of our sample would permit the assessment of these possibilities. Alone, the three dysregulated miRNAs identified in the present study would not represent a satisfactory biomarker for BD. However, the fact that altered miRNA expression can be detected in the blood prior to the onset of BD suggests that further work may one day result in the identification of blood-based biomarkers to aid in the prediction and diagnosis of psychiatric illness, as well as in the monitoring of treatment response. Moreover, it is possible that some blood-expressed molecules will be found to represent good biomarkers for BD despite not being expressed in the brain.

Providing that our findings withstand replication, it would be of great interest to characterise the expression levels of miR-15b, miR-132 and miR-652 in members of the high-risk group who go on to develop psychiatric illness. A key aim of future studies will be to establish whether we can detect differences in gene expression that are predictive of the onset of illness, or indeed differences in gene expression that might suggest mechanisms of resilience. Moreover, characterisation of the downstream pathways affected by the altered expression of these miRNAs is essential in order to build a mechanistic understanding of the biological underpinnings of genetic risk for BD.

## Role of the funding source

This work was supported by a grant from the Chief Scientist Office of Scotland (project grant ETM/181) to KLE, AMM, and DJP. The funders played no role in the study design, in the collection, analysis and interpretation of data, the writing of the report, or in the decision to submit the article for publication.

## Contributors

KLE, RMW, DJP & AMM conceived of the study.

KLE & RMW supervised the execution of the study.

AMM conceived of and supervised the Bipolar Family Study.

SMA, HST & JR extracted RNA, prepared cDNA and carried out qRT-PCR.

RMW & SMA analysed the qRT-PCR data. RMW & RB carried out pathway analysis.

RMW & KLE drafted the manuscript. All authors critically revised the manuscript and approved it for publication.

JES recruited and clinically assessed the participants in the study.

## Conflict of interest

None of the authors declare any potential conflicts of interest.

## Figures and Tables

**Table 1:** Demographic characteristics of the sample.

	Control	High-risk	Test statistic	*p*-value
Age (SD)	26.2 (4.24)	24.9 (4.98)	−1.23	0.224
Gender				
Female (%)	26 (56.5)	18 (52.9)	NA	0.707
Male (%)	20 (43.5)	16 (47.1)		
RIN (SD)	7.05 (0.915)	7.09 (0.859)	0.211	0.833

**Table 2:** Comparison of miRNA expression levels in whole-blood samples from high-risk and control individuals. Nominally significant (*p* ≤ 0.05) *p*-values are indicated in bold.

microRNA	Median expression (IQR)		Mann–Whitney U statistic	*p*-value (uncorrected)	*p*-value (Bonferroni-corrected)	Fold-change
	High-risk	Control				
let-7b	0.953 (0.696–1.35)	0.919 (0.631–1.29)	523	0.605	1.00	1.04
let-7c	0.834 (0.644–1.13)	0.877 (0.583–1.18)	555	0.965	1.00	−1.05
miR-15b	1.07 (0.744–1.42)	0.825 (0.440–0.951)	373	**0.0166**	0.266	1.29
miR-34a	0.637 (0.346–0.942)	0.630 (0.348–0.856)	468	0.737	1.00	1.01
miR-132	1.04 (0.830–1.17)	0.777 (0.651–1.08)	385	**0.0249**	0.398	1.34
miR-133b	0.682 (0.307–1.21)	0.612 (0.231–1.13)	444	0.504	1.00	1.12
miR-134	0.657 (0.397–0.876)	0.576 (0.263–0.970)	489	0.808	1.00	1.14
miR-145	0.777 (0.399–1.16)	0.623 (0.421–0.962)	495	0.485	1.00	1.25
miR-195	0.828 (0.639–1.18)	0.738 (0.589–1.09)	476	0.272	1.00	1.12
miR-212	0.861 (0.780–1.37)	0.946 (0.640–1.31)	500	0.778	1.00	−1.10
miR-221	0.820 (0.673–1.08)	0.734 (0.566–0.906)	430	0.0943	1.00	1.12
miR-432	0.832 (0.549–1.26)	0.826 (0.438–1.30)	538	0.739	1.00	1.01
miR-449	0.734 (0.439–1.13)	0.806 (0.534–1.40)	566	0.317	1.00	−1.10
miR-548d-5p	1.21 (0.783–2.17)	1.33 (0.701–1.90)	484	0.907	1.00	−1.10
miR-572	1.05 (0.791–1.20)	0.876 (0.716–1.06)	442	0.226	1.00	1.20
miR-652	0.898 (0.725–1.21)	0.712 (0.567–1.02)	361	**0.01076**	0.172	1.26

**Table 3:** Summary of the top findings from Ingenuity Pathway Analysis of the mRNA targets of miR-15b.

Name	*p*-value	*q*-value	Ratio
**Canonical pathways**			
Glioblastoma multiforme signalling	9.03 × 10^−8^	2.76 × 10^−5^	36/146 (0.247)
PTEN signalling	1.51 × 10^−7^	2.76 × 10^−5^	31/118 (0.263)
STAT3 signalling	1.75 × 10^−7^	2.76 × 10^−5^	23/73 (0.315)
TGF-β signalling	3.78 × 10^−7^	3.90 × 10^−5^	25/87 (0.287)
PI3K/Akt signalling	4.13 × 10^−7^	3.90 × 10^−5^	31/123 (0.252)

Name	*p*-value range	*q*-value range	No. molecules
**Molecular and cellular functions**			
Cell cycle	5.36 × 10^−17^–4.34 × 10^−4^	1.13 × 10^−13^–1.50 × 10^−2^	278
Cellular assembly and organization	1.16 × 10^−15^–4.29 × 10^−4^	2.20 × 10^−12^–1.50 × 10^−2^	385
Cellular function and maintenance	1.16 × 10^−15^–4.34 × 10^−4^	2.20 × 10^−12^–1.50 × 10^−2^	477
Cell death and survival	1.29 × 10^−15^–3.91 × 10^−4^	2.20 × 10^−12^–1.41 × 10^−2^	613
Cellular growth and proliferation	1.02 × 10^−14^–4.27 × 10^−4^	1.17 × 10^−11^–1.49 × 10^−2^	621
**Physiological system development and function**			
Organismal survival	9.87 × 10^−15^–1.73 × 10^−8^	1.17 × 10^−11^–3.29 × 10^−6^	425
Tissue development	2.15 × 10^−13^–4.27 × 10^−4^	1.75 × 10^−10^–1.49 × 10^−2^	596
Embryonic development	6.22 × 10^−12^–4.12 × 10^−4^	3.04 × 10^−9^–1.46 × 10^−2^	487
Organismal development	6.22 × 10^−12^–4.29 × 10^−4^	3.04 × 10^−9^–1.40 × 10^−2^	598
Nervous system development and function	5.50 × 10^−11^–4.34 × 10^−4^	1.92 × 10^−8^–1.50 × 10^−2^	349
**Diseases and disorders**			
Cancer	1.26 × 10^−26^–4.00 × 10^−4^	2.43 × 10^−22^–1.42 × 10^−2^	1522
Organismal injury and abnormalities	2.95 × 10^−16^–4.41 × 10^−4^	6.32 × 10^−13^–1.51 × 10^−2^	886
Reproductive system disease	2.95 × 10^−16^–3.91 × 10^−4^	6.32 × 10^−13^–1.41 × 10^−2^	735
Gastrointestinal disease	4.28 × 10^−12^–1.00 × 10^−4^	2.22 × 10^−9^–5.29 × 10^−3^	708
Developmental disorder	2.41 × 10^−8^–3.91 × 10^−4^	4.38 × 10^−6^–1.41 × 10^−2^	297

**Table 4:** Summary of the top findings from Ingenuity Pathway Analysis of the mRNA targets of miR-132.

Name	*p*-value	*q*-value	Ratio
**Canonical pathways**			
Molecular mechanisms of cancer	8.57 × 10^−8^	3.36 × 10^−5^	36/365 (0.099)
Axonal guidance signalling	7.03 × 10^−7^	1.38 × 10^−4^	38/432 (0.088)
PI3K/AKT signalling	1.17 × 10^−5^	1.53 × 10^−3^	16/123 (0.13)
Melanocyte development and pigmentation signalling	5.65 × 10^−5^	5.54 × 10^−3^	12/84 (0.143)
B cell receptor signalling	9.12 × 10^−5^	7.01 × 10^−3^	18/176 (0.102)

Name	*p*-value range	*q*-value range	No. molecules
**Molecular and cellular functions**			
Gene expression	7.67 × 10^−13^–1.99 × 10^−3^	2.48 × 10^−9^–4.48 × 10^−2^	180
Cellular growth and proliferation	9.47 × 10^−11^–2.81 × 10^−3^	1.02 × 10^−7^–5.49 × 10^−2^	257
Cellular development	2.10 × 10^−10^–2.83 × 10^−3^	1.70 × 10^−7^–5.49 × 10^−2^	251
Cell death and survival	7.76 × 10^−9^–2.72 × 10^−3^	1.18 × 10^−6^–5.37 × 10^−2^	231
Cell cycle	4.16 × 10^−8^–2.75 × 10^−3^	1.30 × 10^−5^–5.41 × 10^−2^	114
**Physiological system development and function**			
Organismal survival	3.37 × 10^−13^–2.68 × 10^−7^	2.48 × 10^−9^–6.35 × 10^−5^	190
Embryonic development	2.46 × 10^−9^–2.83 × 10^−3^	1.14 × 10^−6^–5.49 × 10^−2^	190
Organismal development	2.46 × 10^−9^–2.72 × 10^−3^	1.14 × 10^−6^–5.37 × 10^−2^	230
Tissue morphology	8.31 × 10^−9^–2.83 × 10^−3^	3.13 × 10^−6^–5.49 × 10^−2^	158
Nervous system development and function	4.98 × 10^−8^–2.83 × 10^−3^	1.42 × 10^−5^–5.49 × 10^−2^	172
**Diseases and disorders**			
Cancer	6.91 × 10^−11^–2.80 × 10^−3^	9.03 × 10^−8^–5.49 × 10^−2^	574
Gastrointestinal disease	6.91 × 10^−11^–2.61 × 10^−3^	9.03 × 10^−8^–5.25 × 10^−2^	305
Developmental disorder	7.24 × 10^−8^–2.33 × 10^−3^	1.95 × 10^−5^–4.95 × 10^−2^	155
Neurological disease	1.23 × 10^−7^–2.71 × 10^−3^	3.03 × 10^−5^–5.37 × 10^−2^	172
Organismal injury and abnormalities	1.27 × 10^−6^–2.83 × 10^−3^	2.30 × 10^−4^–5.49 × 10^−2^	282

**Table 5:** Summary of the top findings from Ingenuity Pathway Analysis of the mRNA targets of miR-652.

Name	*p*-value	*q*-value	Ratio
**Canonical pathways**			
Biotin-carboxyl carrier protein assembly	5.20 × 10^−4^	6.84 × 10^−2^	2/4 (0.5)
Reelin signalling in neurons	8.88 × 10^−4^	6.84 × 10^−2^	5/79 (0.063)
GABA receptor signalling	3.64 × 10^−3^	1.87 × 10^−1^	4/67 (0.06)
Heparan sulfate biosynthesis (Late stages)	1.16 × 10^−2^	3.91 × 10^−1^	3/50 (0.06)
1D-myo-inositol hexakisphosphate biosynthesis II (Mammalian)	1.35 × 10^−2^	3.91 × 10^−1^	2/19 (0.105)

Name	*p*-value range	*q*-value range	No. molecules
**Molecular and cellular functions**			
Cell morphology	8.77 × 10^−5^–4.61 × 10^−2^	1.34 × 10^−1^–2.09 × 10^−1^	36
Cellular assembly and organization	1.43 × 10^−4^–4.61 × 10^−2^	1.34 × 10^−1^–2.09 × 10^−1^	47
Cellular function and maintenance	1.43 × 10^−4^–4.61 × 10^−2^	1.34 × 10^−1^–2.09 × 10^−1^	38
Gene expression	8.31 × 10^−4^–2.69 × 10^−2^	1.38 × 10^−1^–1.80 × 10^−1^	37
Lipid metabolism	9.95 × 10^−4^–4.61 × 10^−2^	1.38 × 10^−1^–2.09 × 10^−1^	11
**Physiological system development and function**			
Tissue morphology	1.28 × 10^−3^–4.61 × 10^−2^	1.38 × 10^−1^–2.09 × 10^−1^	25
Skeletal and muscular system development and function	1.88 × 10^−3^–4.22 × 10^−2^	1.38 × 10^−1^–2.09 × 10^−1^	13
Behaviour	2.02 × 10^−3^–4.61 × 10^−2^	1.38 × 10^−1^–2.09 × 10^−1^	6
Haematological system development and function	3.76 × 10^−3^–4.61 × 10^−2^	1.38 × 10^−1^–2.09 × 10^−1^	11
Immune cell trafficking	3.76 × 10^−3^–3.70 × 10^−2^	1.38 × 10^−1^–1.93 × 10^−1^	5
**Diseases and disorders**			
Cancer	2.61 × 10^−4^–4.61 × 10^−2^	1.38 × 10^−1^–2.09 × 10^−1^	55
Dermatological diseases and conditions	2.61 × 10^−4^–2.79 × 10^−2^	1.38 × 10^−1^–1.80 × 10^−1^	7
Developmental disorder	2.61 × 10^−4^–4.61 × 10^−2^	1.38 × 10^−1^–2.09 × 10^−1^	18
Hereditary disorder	2.61 × 10^−4^–3.88 × 10^−2^	1.38 × 10^−1^–1.97 × 10^−1^	31
Neurological disease	2.61 × 10^−4^–4.61 × 10^−2^	1.38 × 10^−1^–2.09 × 10^−1^	39
