# Supplementary material for: Preliminary investigation of miRNA expression in individuals at high familial risk of bipolar disorder
Source: J Psychiatr Res. 2015 Mar;62:48–55. doi: 10.1016/j.jpsychires.2015.01.006 (PMC4379383; doi:10.1016/j.jpsychires.2015.01.006)
Supplement: Supplementary file 1 [file mmc1.docx]

| **miRNA/small RNA** | **Assay Name** | **Cat. No.** |
| --- | --- | --- |
| let-7b | hsa-let-7b | 002619 |
| let-7c | hsa-let-7c | 000379 |
| miR-15a | hsa-miR-15a | 000389 |
| miR-15b | hsa-miR-15b | 000390 |
| miR-132 | hsa-miR-132 | 000457 |
| miR-652 | hsa-miR-652 | 002352 |
| miR-572 | hsa-miR-572 | 001614 |
| miR-432 | hsa-miR-432 | 001026 |
| miR-221 | hsa-miR-221 | 000524 |
| miR-195 | hsa-miR-195 | 000494 |
| miR-145 | hsa-miR-145 | 002278 |
| miR-133b | hsa-miR-133b | 002247 |
| miR-134 | mmu-miR-134 | 001186 |
| miR-34a | hsa-miR-34a | 000426 |
| miR-449a | hsa-miR-449a | 001030 |
| miR-564 | hsa-miR-564 | 001531 |
| miR-548d-5p | hsa-miR-548d-5p | 002237 |
| miR-548d-3p | hsa-miR-548d-3p | 001605 |
| miR-154* | hsa-miR-154-3p | 000478 |
| miR-212 | hsa-miR-212 | 000515 |
| RNU6B | RNU6B | 001093 |
| RNU47 | RNU47 | 001223 |
| SNORD96A | CS5IO0I | Custom assay |
| SNORD61 | CS6RM6Q | Custom assay |
| SNORD68 | CS70LCY | Custom assay |
| SNORD95 | CS89JI6 | Custom assay |
| SNORD72 | CSAAYOZ | Custom assay |

**SOM table 1.** Details of TaqMan miRNA and small RNA assays used. For each RNA, the assay name and catalogue number are provided.

| **Sample Name** | **Group** | **RIN** |
| --- | --- | --- |
| B016 | Control | 7.6 |
| B021 | Control | 6.3 |
| B034 | Control | 5.9 |
| B044 | Control | 6.3 |
| B045 | Control | 6 |
| B057 | Control | 7.6 |
| B060 | Control | 8.2 |
| B064 | Control | 6.7 |
| B079 | Control | 6.3 |
| B086 | Control | 7.2 |
| B088 | Control | 7.7 |
| B091 | Control | 7.5 |
| B095 | Control | 7.7 |
| B101 | Control | 5.7 |
| B103 | Control | 8.1 |
| B111 | Control | 5.4 |
| B112 | Control | 8.7 |
| B113 | Control | 6.8 |
| B127 | Control | 7.1 |
| B136 | Control | 5.7 |
| B137 | Control | 7.7 |
| B182 | Control | 7.1 |
| B198 | Control | 7 |
| B216 | Control | 6.8 |
| B225 | Control | 7.6 |
| B228 | Control | 8 |
| B231 | Control | 8.1 |
| B239 | Control | 4.5 |
| B251 | Control | 7.1 |
| B269 | Control | 7.1 |
| B272 | Control | 6.6 |
| B274 | Control | 8.7 |
| B279 | Control | 8.5 |
| B280 | Control | 8.1 |
| B287 | Control | 7.6 |
| B290 | Control | 7.5 |
| B291 | Control | 7 |
| B292 | Control | 6.8 |
| B294 | Control | 6.1 |
| G1459 | Control | 7.9 |
| G1463 | Control | 7 |
| G1560 | Control | 5.6 |
| G1756 | Control | 6.6 |
| G1797 | Control | 7.1 |
| G1798 | Control | 6.9 |
| G1799 | Control | 6.6 |
| B001 | High-risk | 7.2 |
| B004 | High-risk | 7.8 |
| B006 | High-risk | 7.6 |
| B025 | High-risk | 5.7 |
| B030 | High-risk | 7.5 |
| B032 | High-risk | 6.6 |
| B036 | High-risk | 5.2 |
| B042 | High-risk | 7.3 |
| B065 | High-risk | 7.5 |
| B069 | High-risk | 8.2 |
| B070 | High-risk | 5.9 |
| B078 | High-risk | 6.5 |
| B094 | High-risk | 8.6 |
| B099 | High-risk | 6 |
| B117 | High-risk | 5.8 |
| B124 | High-risk | 7.5 |
| B128 | High-risk | 5.3 |
| B134 | High-risk | 7.9 |
| B140 | High-risk | 6.8 |
| B145 | High-risk | 7.4 |
| B147 | High-risk | 8.1 |
| B175 | High-risk | 7 |
| B203 | High-risk | 7.8 |
| B205 | High-risk | 8.1 |
| B207 | High-risk | 7.2 |
| B221 | High-risk | 6.2 |
| B240 | High-risk | 6.4 |
| B242 | High-risk | 6.8 |
| B258 | High-risk | 7.6 |
| B266 | High-risk | 7.4 |
| B281 | High-risk | 7.4 |
| B283 | High-risk | 7.4 |
| B288 | High-risk | 7.8 |
| B293 | High-risk | 7.5 |

**SOM table 2.** RNA Integrity scores. Group (control or high-risk) and RNA integrity number (RIN), as measured by the Agilent Bioanalyzer, are shown.

| **miRNA** | **Study details** | **Evidence and additional information** | **References** |
| --- | --- | --- | --- |
| miR-145 | 667 miRNAs analysed in the PM PFC of SCZ and BD patients (both n=35), confirmed by single-tube real-time PCR^1^ | Increased expression in BD patients^1^ | ^1^Kim, A. H., Reimers, M., et al. (2010). Schizophr Res, 124(1-3), 183-191 |
| miR-154*, miR-133b, miR-212, miR-132 | 667 miRNAs analysed in the PM PFC of SCZ and BD patients (both n=35), confirmed by single-tube real-time PCR^1^ | Dysregulated expression BD and SCZ patients^1^. The expression of miR-132 and miR-212 is dysregulated in Alzheimer's Disease, highlighting involvement in neuropsychiatric disease^2^. | ^1^Kim, A. H., Reimers, M., et al. (2010). Schizophr Res, 124(1-3), 183-191, ^2^Cogswell, J. P., Ward, J., et al. (2008). J Alzheimers Dis, 14(1), 27-41 |
| miR-134 | Analysis of miR-134 plasma expression in BD patients (n=21). Assessment of the effect of antipsychotic medication on miR-134 expression^3^ | Decreased expression in drug-free patients. Expression in patients was increased following treatment with typical antipsychotics^3^ | ^3^Rong, H., Liu, T. B., et al. (2010). J Psychiatr Res, 45(1), 92-95 |
| miR-15a | 124 miRNAs assayed in the PM STG (n=21) and DLPFC (n=15) of SCZ patients, confirmed by quantitative real-time PCR^4^ 435 miRNAs assayed in the PM DLPFC of BD and SCZ patients (both n=35)^5^ | Dysregulated expression in BD and SCZ patients^4,5^. Family member miR-15b also found to show dysregulated expression^4,6^. These two miRNAs share the same seed-pairing region, and therefore a high proportion of target genes. | ^4^Beveridge, N. J., Gardiner, E., et al. (2010). Mol Psychiatry, 15(12), 1176-1189, ^5^Moreau, M. P., Bruse, S. E., et al. (2011). Biol Psychiatry, 69(2), 188-193, ^6^Perkins, D. O., Jeffries, C. D., et al. (2007). Genome Biol, 8(2) |
| miR-34a | 667 miRNAs analysed in the PM PFC of SCZ and BD patients (both n=35), confirmed by single-tube real-time PCR^1^ 365 miRNAs assayed in the mononuclear leukocytes of SCZ patients (n=30), followed by validation in an independent sample of 30 controls and 60 SCZ patients^7^ 13 miRNAs in 20 lymphoblastoid cell lines assayed following treatment with lithium for 4, 8, and 16 days^8^ Microarray to identify miRNAs differentiatlly expressed in rat hippocampus following chronic treatment with lithium and valproate, confirmed by quantitative real-time PCR^9^ | miR-34a shows altered expression in the brain and blood of BD and SCZ patients, and its expression is affected by the mood stabalising drugs lithium and valproate ^1,7,8,9^. It forms part of a 7 miRNA gene expression signature detected in the blood that can discriminate between SCZ cases and controls^7^. | ^1^Kim, A. H., Reimers, M., et al. (2010). Schizophr Res, 124(1-3), 183-191, ^7^Lai, C. Y., Yu, S. L., et al. (2011). PLoS One, 6(6), ^8^Chen, H., Wang, N., et al. (2009). Int J Neuropsychopharmacol, 12(7), 975-981, ^9^Zhou, R., Yuan, P., et al. (2009). Neuropsychopharmacology, 34(6), 1395-1405 |
| miR-449a, miR-432, miR-572, miR-652, miR-564, miR-548d | 365 miRNAs assayed in the mononuclear leukocytes of SCZ patients (n=30), followed by validation in an independent sample of 30 controls and 60 SCZ patients.^7^ | Together with miR-34a, the miRNAs form a 7 miRNA gene expression signature identified in the blood that can discriminate between SCZ patients and controls^7^ | ^7^Lai, C. Y., Yu, S. L., et al. (2011). PLoS One, 6(6) |
| miR-15b | 124 miRNAs assayed in the PM STG (n=21) and DLPFC (n=15) of SCZ patients, confirmed by quantitative real-time PCR (STG: n=21; DLPFC: n=15)^4^ | Increased expression in SCZ patients^4^. Family member miR-15a also found to show dysregulated expression. These two miRNAs share the same seed-pairing region, and therefore a high proportion of target genes^4,5^ | ^4^Beveridge, N. J., Gardiner, E., et al. (2010). Mol Psychiatry, 15(12), 1176-1189, ^5^Moreau, M. P., Bruse, S. E., et al. (2011). Biol Psychiatry, 69(2), 188-193 |
| miR-195 | 264 miRNAs assayed in the PM PFC of patients with SCZ (n=13) or schizoaffective disorder (n=2), followed by confirmation by quantitative real-time PCR (n=4)^6^ Analysis of miR-195 expression in the PM PFC of SCZ patients (n=20)^13^ | Decreased expression in SCZ (and schizoaffective disorder) patients^6,13^. Previously shown to regulate BDNF, which regulates GABA-ergic genes found dysregulated in schizophrenia^11^. Shares a seed pairing region with miR-15a and -15b, and therefore a high proportion of target genes. | ^6^Perkins, D. O., Jeffries, C. D., et al. (2007). Genome Biol, 8(2), ^10^Mellios, N., Huang, H. S., et al. (2009). Biol Psychiatry, 65(12), 1006-1014, ^11^Mellios, N., Huang, H. S., et al. (2008). Hum Mol Genet, 17(19), 3030-3042 |
| miR-let-7b, miR-let-7c | Microarray to identify miRNAs differentiatlly expressed in rat hippocampus following chronic treatment with lithium and valproate, confirmed by quantitative real-time PCR^9^ | Decreased expression in rat HPC followig long-term treatment with lithium or valporate^9^. Family member miR-let-7d, which shares the same see-pairing region, has been found to show increased expression in brains of SCZ patients^4^, suggesting dysregulation of let-7 targets as a pathogenic mechanism. | ^4^Beveridge, N. J., Gardiner, E., et al. (2010). Mol Psychiatry, 15(12), 1176-1189, ^9^Zhou, R., Yuan, P., et al. (2009). Neuropsychopharmacology, 34(6), 1395-1405 |
| miR-221 | 13 miRNAs assayed in 20 lymphoblastoid cell lines following treatment with lithium for 4, 8, and 16 days^8^ Microarray to identify miRNAs differentiatlly expressed in rat hippocampus following treatment with lithium and valproate, confirmed by quantitative real-time PCR^9^ | Altered expression following treatment with mood stabalisers in rat HPC and human lymphoblastoid cell lines^8,9^ | ^8^Chen, H., Wang, N., et al. (2009). Int J Neuropsychopharmacol, 12(7), 975-981, ^9^Zhou, R., Yuan, P., et al. (2009). Neuropsychopharmacology, 34(6), 1395-1405 |

**SOM table 3.** miRNAs selected for measurement by qRT-PCR in individuals genetically at high-risk of BD. Shown are details of previous studies implicating these miRNAs in the pathogenesis of BD or SCZ. Abbreviations: BD: bipolar disorder; SCZ: schizophrenia; PM: post-mortem; HPC: hippocampus; PFC: prefrontal cortex; DLPFC: dorsolateral prefrontal cortex; STG: superior temporal gyrus
